# Assays of different aspects of haemostasis – what do they measure?

**DOI:** 10.1186/s12959-015-0036-2

**Published:** 2015-02-05

**Authors:** Nahreen Tynngård, Tomas L Lindahl, Sofia Ramström

**Affiliations:** Department of Clinical Chemistry, and Department of Clinical and Experimental Medicine, Linköping University, Linköping, Sweden; Department of Clinical Immunology and Transfusion Medicine, and Department of Clinical and Experimental Medicine, Linköping University, Linköping, Sweden; Department of Clinical and Experimental Medicine, Linköping University, Linköping, Sweden

**Keywords:** Coagulation, Haemostasis, Platelets, Coagulation assays, Platelet function testing

## Abstract

Haemostasis is a complex process affected by many factors including both cellular and plasma components. It is a multistep process starting with platelet adhesion to damaged endothelium and ending in clot fibrinolysis. There are several methods available to study different aspects of haemostasis including adhesion, aggregation, coagulation and fibrinolysis. This review describes the different methods, what aspects of haemostasis they measure and their limitations. Methods discussed include methods to study adhesion (e.g. PFA-100, cone and platelet(let) analyzer and perfusion chambers) and aggregation (e.g. Multiplate, VerifyNow and Plateletworks). Furthermore the principles behind viscoelastic haemostatic assays are presented as well as methods that can analyse aspects of haemostasis in plasma or platelet-rich-plasma samples (thrombin generation, overall haemostasis potential and Thrombodynamics Analyzer).

## Introduction

Haemostasis is a complex process involving adhesion of platelets to damaged endothelium, formation of a platelet plug (aggregation), formation of a fibrin network to stabilise the plug, clot retraction and finally fibrinolysis. In addition all of these processes take place under flow and is controlled and regulated by factors released from the surrounding endothelium. Surgery may cause coagulopathy due to acidosis, hypothermia and haemodilution and is associated with increased fibrinolytic activity which warrants monitoring to guide transfusion therapy [1,2].

Thus an “ideal” haemostasis assay should preferably measure all these processes at relevant shear conditions and provide results in a timely manner. Indeed, there are many different methods available for measuring one or more of the many diverse events of the haemostatic process and platelet function. However, none of the methods currently available measures all these processes and they all have their pros and cons. This review will describe a number of assays used for evaluation of different aspects of haemostasis from a technical point of view, highlighting their methodology and what they can and cannot measure, with conclusions summarized in Table 1.

**Table 1:** **Aspects of global haemostasis assays**

**Assay**	**Sample type**			**Adhesion**	**Aggregation**	**Coagulation**				**Endothelium**	**Shear**	**No. of citations**
	Plasma	PRP	WB			Init.	Prop.	Elast.	Lysis			
Multiplate	**-**	**+**	**+**	**-**	**+**	**-**	**-**	**-**	**-**	**-**	**-**	250
VerifyNow	**-**	**-**	**+**	**-**	**+**	**-**	**-**	**-**	**-**	**-**	**-**	476
Plateletworks	**-**	**-**	**+**	**-**	**+**	**-**	**-**	**-**	**-**	-	**-**	26
Impact-R	**-**	**-**	**+**	**+**	**+**	**-**	**-**	**-**	**-**	**-**	**+**	121
PFA-100	**-**	**-**	**+**	**+**	**+**	**-**	**-**	**-**	**-**	**-**	**+**	745
Perfusion chambers	**-**	**-**	**+**	**+**	**+**	(+)	(+)	**-**	**-**	(+)	**+**	630
TEG	(+)	**+**	**+**	**-**	**-**	**-**	**+**	**+**	**+**	**-**	**-**	4016
ROTEM	(+)	**+**	**+**	**-**	**-**	**-**	**+**	**+**	**+**	**-**	**-**	3932
ReoRox	(+)	**+**	**+**	**-**	**-**	**+**	**+**	**+**	**+**	**-**	**-**	28
Sonoclot	(+)	**+**	**+**	**-**	**-**	**+**	**+**	**+**	**+**	**-**	**-**	113
Thrombin generation	**+**	**+**	**−**	**-**	**-**	**+**	**+**	**−**	**−**	**-**	**-**	118
OHP	**+**	**−**	**−**	**-**	**-**	+	(+)	**-**	**+**	**-**	**-**	23
Thrombodynamics	**+**	**+**	**+**	**-**	**-**	**+**	**+**	**−**	**−**	**-**	**-**	11

The assays ability to measure adhesion, aggregation, coagulation in terms of initiation (Init.), propagation (Prop.), clot elasticity (Elast) and fibrinolysis (Lysis). The table shows the type of sample (plasma, platelet-rich-plasma (PRP) or whole blood (WB)) that can be assessed in each assay. The table also shows if the measurement can include the contribution by endothelium and shear components. Plus (+) means yes and minus (−) means no, signs within parentheses means possible in theory, but not commonly used. For the propagation component, the perfusion chambers and the Thrombodynamics detects spatial clot propagation, whereas the other techniques detects other processes occurring after the initial clotting and first fibrin fibres have formed as propagation. To reflect how common these different assays are, we present the number of citations found on PubMed on the 30^th^ of November 2014.

### Tests of primary haemostasis

#### Aggregometry

In addition to the classical light transmission aggregometry that requires preparation of platelet-rich plasma, thus limiting its usefulness in the acute clinical setting, commercial tests for assessment of aggregation in whole blood have been launched. In general, the tests have been designed to monitor treatment response to the common classes of anti-platelet drugs, aspirin, P2Y_12_ (ADP- receptor) - antagonists and GPIIb/IIIa antagonists by adding arachidonic acid, ADP or a strong platelet agonist such as the thrombin PAR1 receptor-activating peptide (TRAP, amino acid sequence SFLLRN) or collagen, respectively.

In the Multiplate® (Roche, Switzerland) [3] the blood sample (anticoagulated with citrate or hirudin) is added to a disposable cuvette containing electrodes to which platelets adhere and aggregate following addition of collagen, TRAP, ADP, arachidonic acid or ristocetin. This causes a change in impedance which is registered. Multiplate is designed to measure aggregation in whole blood but can be used on platelet suspensions [4].

VerifyNow® (Accumetrics, USA) is a fully automated cartridge-based instrument for assessment of antiplatelet medications, with three types of test cartridges, containing ADP, arachidonic acid or TRAP. Citrated whole blood is mixed with the platelet agonist and fibrinogen-coated beads. Activated platelets will bind to the beads and agglutinate and the increase in light transmittance due to this will be recorded [5].

The Plateletworks® (Helena Laboratories, USA) aggregation kits are based upon comparing platelet counts within a control EDTA tube and after aggregation with ADP, arachidonic acid or collagen within citrated tubes. Results are expressed as % aggregation or % inhibition [6].

Over the past years a number of studies have been trying to establish the optimal test and range of platelet reactivity associated with the highest protection against thrombosis and the lowest risk of bleeding. The reported correlation between on-treatment platelet reactivity and outcome also suggested the use of platelet function testing to personalize antiplatelet therapy. Several studies have also been conducted in this field, but major clinical trials have failed to demonstrate a benefit of such a strategy in improving clinical outcomes (recently reviewed by [7,8]).

#### Adhesion assays

The classical adhesion test is to count platelets before and after passage of heparinised blood through a column filled with glass beads. Commercially available instruments include the platelet function analyzer 100 (PFA-100®, Siemens Healthcare Diagnostics Inc., USA) and the cone and plate(let) analyzer (CPA) called Impact-R® (DiaMed, Switzerland). Both of these tests measure platelet adhesion and aggregation under conditions of high shear and require anti-coagulated whole blood [9,10]. The PFA-100 measures the time to occlusion (CT, closure time) of blood flow through a collagen coated membrane in the presence of epinephrine or ADP. In the CPA system the sample is added to a polystyrene well and plasma proteins adheres to the surface of the well. Shear stress is applied by rotating a conical device immersed in the sample, leading to adhesion and aggregation of platelets. The platelets are visualized and quantified by staining. The results are expressed as percentage of surface covered by aggregates (SC) and average aggregate size (AS) [11]. The PFA-100 and the CPA have been shown to detect wWF disorders [3,9,12] and GPIIb/IIIa inhibitors [6,10]. There are also other instruments where shear can be applied, either using the cone-and-plate technology or the coaxial cylinder coquette, where the blood is placed between two coaxial cylinders where the inner one rotates to generate the shear [13].

Platelet adhesion can also be analysed using perfusion chambers [14]. The perfusion system consists of two parallel plates. One of the plates accepts a glass coverslip that can be coated with proteins, e.g. fibrinogen, collagen, vWF, laminin, or with endothelial cells. Platelet surface coverage, aggregate size and platelet rolling velocities can be evaluated by video microscopy or confocal microscopy. The shear can be varied which allows the coagulation process to be studied under physiological flow conditions. Ready-made flow chambers, sometimes also offering integrated pump-, imaging- and/or analysis systems are now entering the market [15]. The use of flow-based assays for assessment of haemostasis has been recently reviewed by the ISTH SSC Biorheology Subcommittee [13,16]. As platelet adhesion in flow-based devices require the presence of red blood cells, platelet suspensions such as platelet concentrates for transfusion cannot be easily evaluated. Therefore, we have recently proposed a novel assay where platelet activation upon adhesion to protein-coated beads can be studied using flow cytometry [17].

### Tests of secondary/global haemostasis

#### Viscoelastic haemostatic assays (VHA)

The viscosity of the blood increases as the fibrin network forms during blood coagulation. Viscoelastic assays enables analysis of clot formation, clot elasticity development and the fibrinolysis process in real time. The elasticity of the clot depends on several factors; the contractile force exerted by the platelets during clot retraction, platelet concentration [18-20], haematocrit [18,21], fibrinogen concentration [18-20,22], FXIII [23,24] and the thrombin generation during coagulation [25]. However, it is important to emphasize that the major signal comes from platelet contractile forces and not from the fibrin network [26,27].

**Figure 1:**
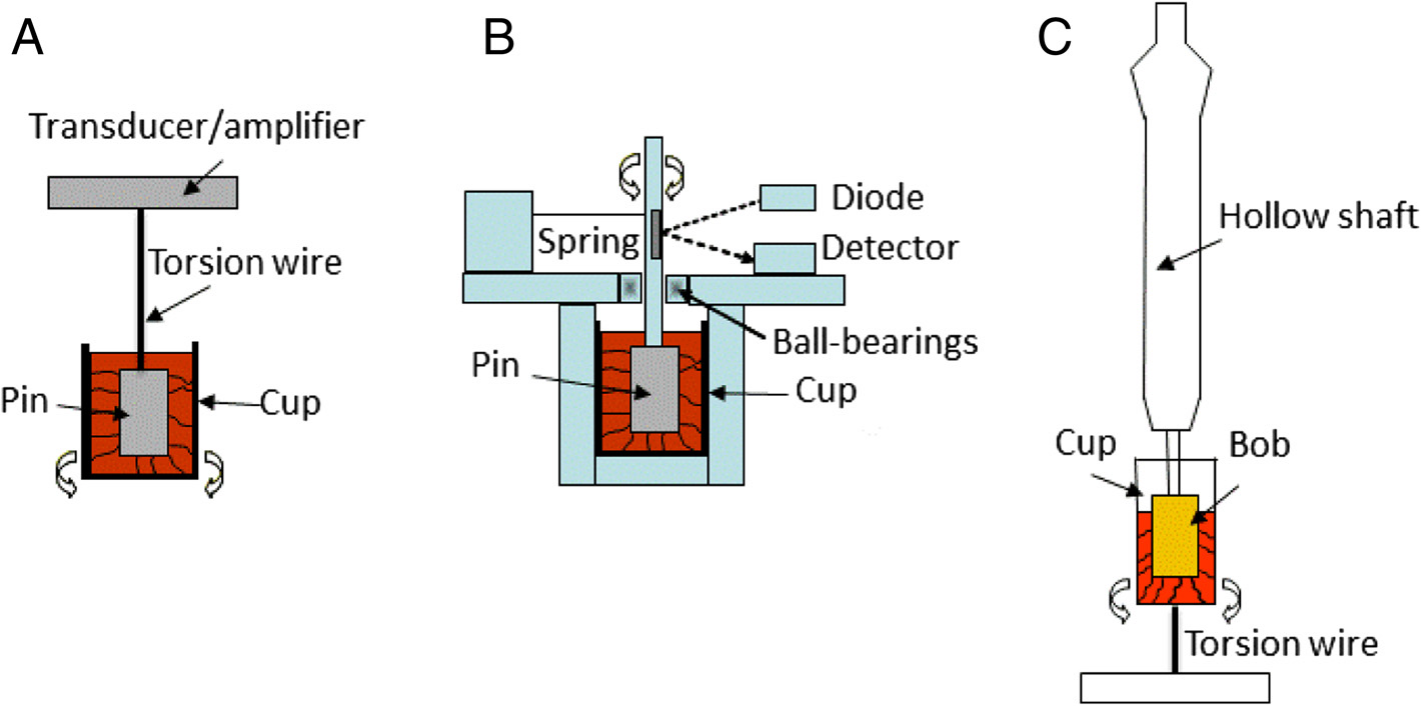
**The measuring principle of TEG (A), ROTEM (B) and ReoRox FOR (C).** In the TEG instrument, a pin is suspended by a wire into a cup containing the blood sample. The cup rotates back and forth 4.75° every 10 seconds. During coagulation of the sample, fibrin strands will form between the pin and the wall of the cup which will affect the movement of the cup which is gradually transmitted to the pin. In the ROTEM instrument, the cup is stationary and a ball-bearing pin rotates back and forth 4.75°. The movement of the pin is driven by an elastic spring. Also here fibrin strands will form between the wall of the cup and the pin during coagulation and the strength of the strands will affect the movement of the pin. In the ReoRox, the cup is turned up every 2.5 seconds and then released, allowing rotational oscillation around the longitudinal axis. An optic sensor records the frequency and damping of the oscillation. A pin (bob) is immersed into the cup via a shaft. The fibrin fibres formed during coagulation will couple the cup to the bob, and the amount and activity of platelets bound to the fibrin network, will affect the frequency and damping of the oscillation.

**Figure 2:**
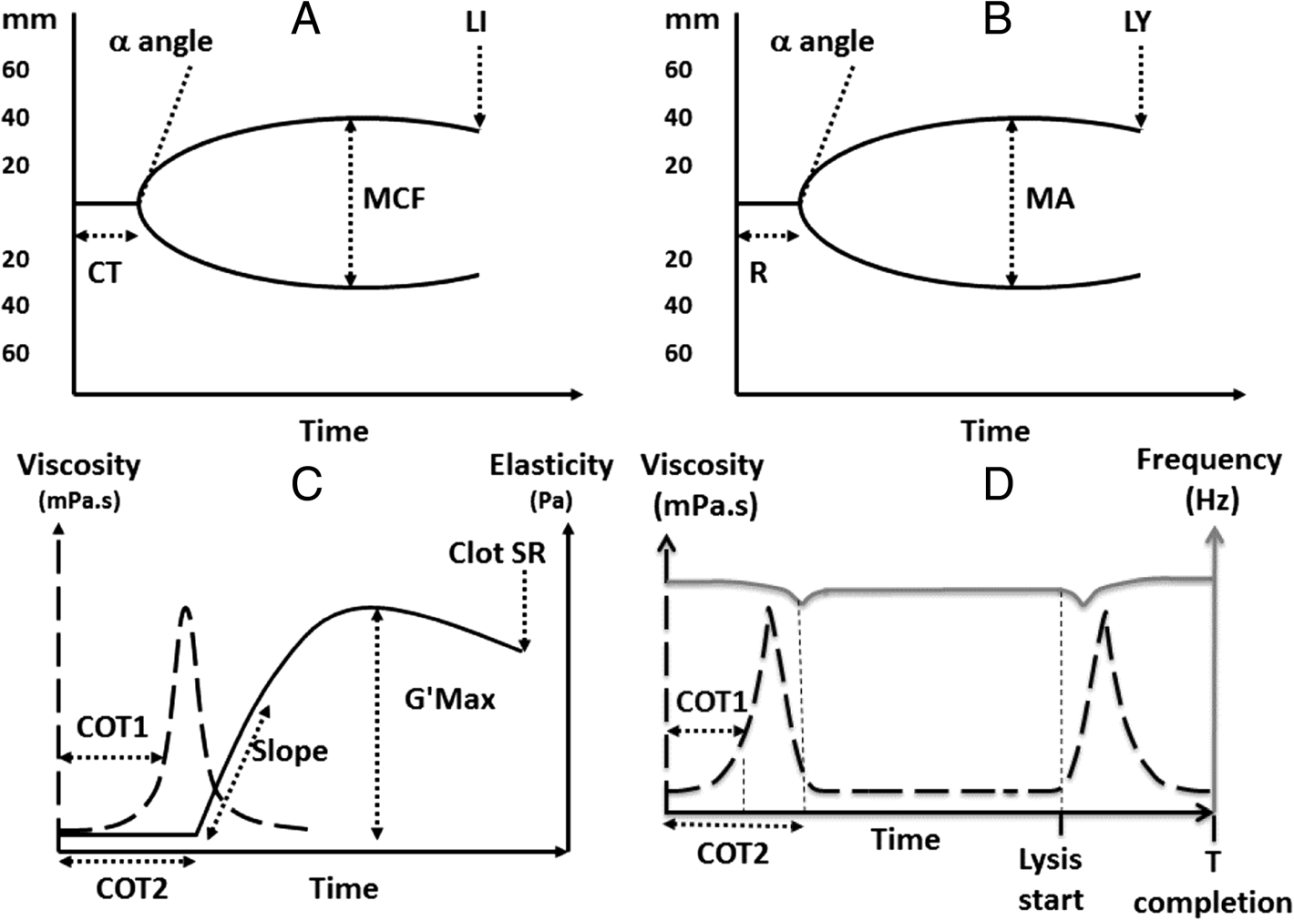
**Tracings from TEG (A), ROTEM (B) and ReoRox (C, D) with analysis variables depicted.** Clotting time as detected by TEG and ROTM (R, CT, respectively) and by ReoRox (COT1 and COT2). The elasticity propagation variable by the instruments (alfa by TEG and ROTEM and Slope by ReoRox). The maximum clot strength (MA by TEG, MCF by ROTEM and G'max by ReoRox). Fibrinolysis variables are LY30 by TEG, LI30 by ROTEM, and Clot SR and Lysis start and T completion (complete fibrinolysis) by ReoRox.

Thromboelastography was first described by Hartert in 1948 [28]. There are two commercial thromboelastographs, TEG® (Haemonetics, USA) and ROTEM® (Pentapharm, Switzerland). The measuring unit of both instruments consists of a cylindrical cup, made of disposable plastic. A pin is suspended into the cup, and the pin is connected to a detector. The cup and the pin will make a forced oscillation relative each other with an angle of 4.75° (Figure 1) [25,29]. It is not possible to measure the blood viscosity or to detect the initiation of coagulation, as the first signals appear when the pin is connected via the first fibrin fibres spanning the whole distance to the wall of the cup. The clot elasticity is expressed in mm in the tracing (Figure 2). The major difference between the instruments lies in the oscillation. In the TEG instrument the cup oscillates and in the ROTEM instrument the pin oscillates. The ROTEM instrument has an electronic pipette connected to the instrument to simplify pipetting of the different reagents provided, and the software provides exact step-by-step instructions to make the instrument easy to use.

**Figure 3:**
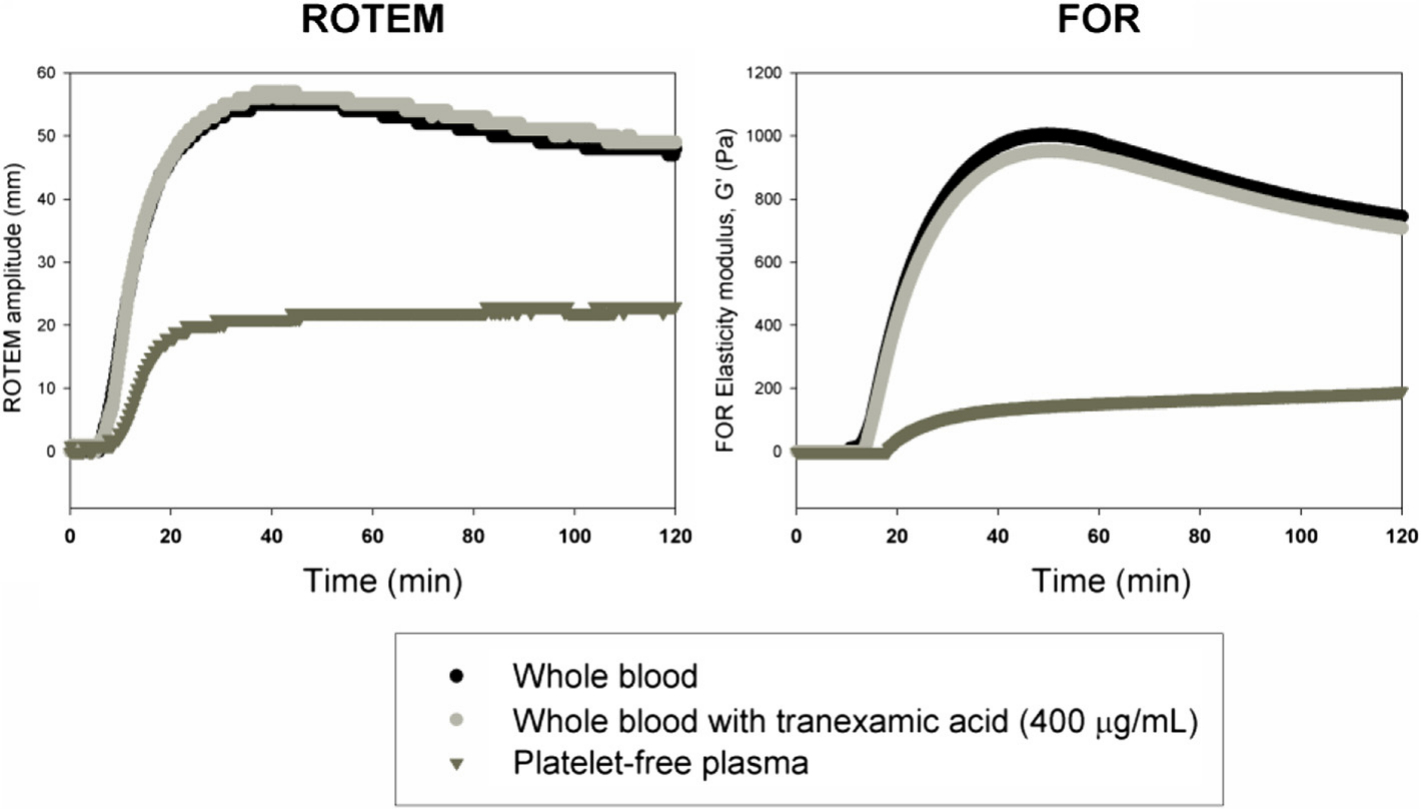
**Contribution of platelets and fibrinolysis to the decrease in ROTEM® or FOR curves.** In normal individuals, the decrease in ROTEM curve amplitude (left) or FOR G’ (right) occur even in the presence of tranexamic acid (Cyklokapron®) in doses capable of preventing fibrinolysis induced by added t-PA (light grey curve). On the contrary, the decrease was platelet-dependent, as it was totally absent in platelet free plasma (PFP, dark grey curve).

Free oscillation rheometry (FOR) (MediRox AB, Sweden) is a newer technology which makes it possible to measure changes in viscosity and elasticity in clotting whole blood and in dissolving clots and to obtain results in SI-units. In this instrument (ReoRox®), oscillation is initiated by a forced turn of the sample cup every 2.5 seconds (Figure 1). After a brief hold time, the sample cup is released, allowing free rotational oscillation around the longitudinal axis. An optic angular sensor records the frequency and damping of the oscillation as a function of time. FOR analysis includes simultaneous measurement of blood viscosity and thus allows detection even of the initial phases of coagulation, before connection of the cup and bob by fibrin fibres. Gold-plating of the cylindrical cup and the bob immersed in the centre is used to ensure strong, covalent binding of fibrinogen to the cup and bob. This prevents detachment when platelet contractile forces are increasing during clot retraction, which has been suggested as a potential problem in TEG and ROTEM [30]. Potential detachment, as well as differences in oscillation and detection strategies may be partial explanations to why the difference between platelet-free plasma and whole blood is smaller in ROTEM than in FOR (see Figure 3).

All VHA systems have a panel of different tests focusing on different aspects of the coagulation process (Table 2). The tests commercially available for TEG use kaolin or a combination of kaolin and tissue factor (TF) to activate intrinsic or extrinsic pathway of coagulation, respectively [31]. ROTEM also has reagents that can activate the intrinsic or extrinsic pathway. Both systems have tests to monitor treatment with heparin and protamine [32] as well as for evaluation of the contribution of fibrin to the clot strength [31,33]. None of the above tests can be used to monitor anti-platelet treatment with ADP-receptor inhibitors or aspirin. The PlateletMapping™ assay was introduced to monitor anti-platelet therapy by TEG. A native blood sample activated by kaolin is compared with heparinised samples where the fibrin network is formed by adding reptilase and FXIIIa and the platelets are activated by ADP or arachidonic acid [34]. This test predicted a first ischemic event after elective stenting in patients with coronary artery disease on clopidogrel and aspirin [35]. TEG has been used to monitor blood component therapy in patients undergoing cardiac, liver, or trauma surgery [36-39]. Treatment algorithms have been developed to interpret the results and guide treatment with blood components [31,33,40]. Low clot strength in trauma patients is associated with increased mortality [39]. For the FOR, there is a panel of tests available including tests to monitor heparin treatment and for evaluation of the contribution of fibrin to the clot strength. The coagulation is activated via the extrinsic pathway or by addition of a specific PAR1-activating peptide in the TRAP test to activate platelets via thrombin receptor PAR1.The instrument has mainly been used for studies of the platelet contribution to whole blood coagulation and quality control of platelet concentrates [18,41-44]. The clinical studies published so far have been limited in number [45-48]. FOR reports a wider measuring range for elasticity as compared to thromboelastography [18,49]. As an example, in pregnancy the increase in clot elasticity was significant already in the first trimester utilising FOR, with an gradual increase to 52% in the third trimester [47], while no significant changes in maximal amplitude between trimesters were detected using TEG, and the maximum increase during pregnancy was less than 8% [50].

**Table 2:** **TEG, ROTEM and ReoRox assays**

**TEG**	**ROTEM**	**ReoRox**
**KaolinTEG**	**InTEM**	**HepScreen1**
Kaolin (intrinsic pathway activation)	Intrinsic pathway activation, sensitive to heparin	TP (extrinsic pathway, sensitive to heparin)
**HepTEG**	**HepTEM**	**HepScreen2**
Heparin neutralising cup	Intrinsic pathway activation + heparin neutralisation	TP (extrinsic pathway + heparin neutralization)
**RapidTEG**	**ExTEM**	**FibScreen1**
Kaolin + TF activation	TF activation (extrinsic pathway)	TP (extrinsic pathway)
**Functional fibrinogen TEG (FFTEG)**	**FibTEM**	**FibScreen2**
TF activation + platelet inhibition with abciximab	TF activation + platelet inhibition with cytochalasin	TP activation + platelet inhibition with abciximab)
	**FibTEM+**	
	TF activation + platelet inhibition with cytochalasin and tirofiban (under development)	
**Platelet Mapping Kit**	**ApTEM**	**ReoTrap**
(ADP, AA)	(TF activation + aprotinin, verification of fibrinolysis)	PAR-1 activation

TEG, ROTEM and ReoRox assays and their respective sensitivities and clot initiating substances. Tissue factor (TF), thromboplastin (TP).

TEG and ReoRox uses abciximab, which blocks the binding of fibrinogen to the GPIIb receptor, to evaluate the contribution of fibrin to the clot strength whereas ROTEM uses cytochalasin D which inhibits the reorganisation of the platelet cytoskeleton. However, it has been shown that these substances alone might not completely block the platelet contribution to the clot strength [26] and that a combination of cytochalasin D and abciximab or tirofiban (similar mechanism of action as abciximab) more effectively blocks platelets [51].

The VHA instruments usually operate at 37°C but the temperature can be adjusted. This allows coagulation to be monitored in patients with hypothermia which is important since hypothermia affects coagulation [24]. Hyperfibrinolysis during trauma or major surgery is an important therapeutic target in bleeding management. VHA can be used to detect fibrinolysis by measuring the reduction in elasticity (Figure 2). However, it is important to recognise that under normal conditions, the “lysis” parameter in these instruments does not reflect ongoing fibrinolytic activity in the sample, but instead mirrors the decrease in platelet contractile force, probably due to exhaustion, as this decrease is also seen in samples treated with the fibrinolysis inhibiting drug tranexamic acid (Figure 3). In addition, the test used in the VHA can influence the ability of the VHA to detect fibrinolysis [52,53]. For example, RapidTEG was shown to be less sensitive than kaolin TEG to low fibrinolytic activity [52]. It has been pointed out that currently available assays to TEG and ROTEM are insufficient to detect low increase in fibrinolytic activity [54]. FOR can also detect increasing fibrinolytic activity by measuring changes in viscosity when the blood reverts back into liquid form (Figure 2) [53]. VHAs have been used to assess the effects of haemodilution with Ringer’s actetate, hydroxylethyl starch (HES) and albumin on coagulation and treatment with fibrinogen and FXIII concentrates in vitro [23,24,27,55,56].

The Sonoclot® analyser (Sienco, Inc., USA) has a tubular probe that oscillates up and down in the whole blood sample. The resistance to movement is measured and recorded during the coagulation process [57]. Since the introduction in the 70’s [58], the use of Sonoclot® has been relatively limited as compared to TEG and ROTEM.

#### Other whole blood tests

A number of other approaches to evaluate “global haemostasis” have been described and commercialised, but not gained widespread interest. However, as it may be interesting to discuss their methodology, we will describe them briefly in this section.

The Clot Signature Analyzer™ (CSA™; Xylum Corporation, USA) is a global haemostasis screening instrument intended for use with native whole blood [59]. The blood is passed through a thin plastic tube under pressure. Two holes are then punched in the tube, causing a fall in pressure. The platelet and fibrin clot will plug the holes and this time is recorded as the “platelet hemostasis time” (PHT). The CSA also records the clot time (CT) as the coagulation spreads though the lumen of the tube. A subsample of the blood is also passed through a second tube with collagen fibrils and the time to reach a 50% pressure reduction in this tube is called collagen-induced thrombus formation time (CITF). Despite a relatively good sensitivity in picking out patients with known bleeding disorders in a multi-centre trial [60], the test could not help in distinguishing between platelet and coagulation factor defects.

The Gorog Thrombosis Test (GTT) [61] or thrombotic status analyser (TSA) [62] (Montrose Diagnostics, UK) adds native blood to a vertical conical tube with a hole in the bottom. The tube also contains two steel balls. Platelets are activated by shear stress (175 dyne/cm^2^) when they pass the first large ball, and will then aggregate and initiate coagulation in the area between the balls. A light sensor records the time between blood droplets leaving the end of the tube to determine the time for occlusion, and the time for thrombolysis when blood flow starts again.

The Hemostasis Analysis System (HAS; Hemodyne, Inc., USA) is measuring the force developed by platelets as they undergo cellular contraction (“platelet contractile force”, PCF™), and speed of clot formation in whole blood between a cup and parallel upper plate at 37°C [63]. The time between assay start and PCF onset is termed the thrombin generation time (TGT™) and is used as a surrogate marker for thrombin generation [64].

The HemoSTATUS™ test or “platelet-activated clotting time” (Medtronic Blood Management, USA) measures acceleration of kaolin-activated clotting time (ACT) by different concentrations of platelet activating factor (PAF). The test has mainly been used for the evaluation of patients during cardiac surgery [65,66], even though its usefulness for predicting blood loss has been questioned [67,68].

## Tests performed in plasma or platelet-rich plasma

### Thrombin generation (CAT assay)

The Thrombinoscope (Thrombinoscope BC, Netherlands) can be used to measure thrombin generation (TG) variables in the presence or absence of platelets. TG is carried out in a 96-well plate fluorometer. The fluorescence of the sample is compared to a calibrator. TG variables include lag-time, maximum thrombin concentration (Cmax), time required to reach Cmax (Tmax) and endogenous thrombin potential (ETP). TG is affected by deficiencies in coagulation factors II, V, VIII, IX and X but to a lesser degree by fibrinogen, FXIII and FVII [69] and the assay is not affected by haemodilution [55].

#### *Overall Hemostasis Potential* (OHP)

The overall hemostasis potential (OHP) is a plasma-based assay based on repeated spectrophotometric registration of the fibrin-aggregation curve in platelet-poor plasma containing small amounts of exogenous thrombin, tissue-type plasminogen activator, and calcium. The overall coagulation potential and overall fibrinolytic potential are supplementary parameters of OHP, with studies reported in a number of hyper- and hypocoagulable states and during anticoagulant treatment (recently reviewed in [70]).

### Propagation of coagulation

A commercial instrument for detection of propagation of coagulation in plasma from a TF-coated surface has been recently introduced and described in several articles [71-73] (Thrombodynamics Analyzer™, HemaCore, Russia). Coagulation is detected in a cuvette by time-lapse image capture of light scattering from the fibrin network. By image processing and analysis, both the initiation (lag time in minutes) and propagation phase (initial rate of clot growth; μm/min) of the coagulation process can be measured in the same experiment. An updated version will also measure thrombin generation in the growing clot utilizing a chromogenic substrate.

## Discussion

Several of the assays described are affected by platelet concentration (e.g. the PFA-100, CPA, VHA’s, and VerifyNow) [5,9,10,18-20]. Multiplate was also shown to be affected by platelet concentration [74] but another study reported only a weak correlation within the normal range [3]. Also haematocrit affect the result of several methods including VHA’s [18,21]. However, the aggregation assays have shown to vary in their sensitivity to haematocrit. The VerifyNow was shown to be affected by haematocrit [75,76] whereas the effect on the Multiplate assay was less pronounced and depended on the agonist used in the assay [21,77] and the Plateletworks was not affected at all [6]. The perfusion chambers and CPA devices require the presence of red blood cells, which complicates the analysis of platelet suspensions or samples with a low haematocrit [11,78,79]. Also the PFA-100 require a haematocrit of >10% [80].

Another difference between the methods is the use and choice of anti-coagulant which can affect the results. Several methods including the PFA-100, CPA and VerifyNow use citrated anti-coagulated blood and thus coagulation is inhibited by chelation of calcium ions [5,75,81]. Several anti-coagulants can be used in the perfusion chambers and might result in different results depending on the choice of anti-coagulant [14]. The VHA’s have the advantage that both non-anti-coagulated whole blood and citrated blood can be used in the assay and coagulation is allowed in the citrated samples by addition of calcium. However, if reagents giving longer clotting times are used, it is important to consider and avoid unintentional contact activation in the blood collection tubes, as this might otherwise affect the test results [82]. The PFA-100 is also affected by the blood group further complicating data interpretations [3]. The recommended maximum time from blood sampling to analysis varies between the methods and may also influence the results and each manufacturer has established recommendations regarding sample stability.

The VHA’s (TEG, ROTEM and ReoRox) have the advantage that they can measure coagulation, platelet function, clot retraction and fibrinolysis simultaneously. However, in contrast to the aggregation assays, VHA are in general insensitive to anti-platelet treatment with aspirin and ADP-receptor inhibitors with the exception of the Platelet Mapping assay [34]. PFA-100 has also shown variable sensitivity to aspirin and variable sensitive to clopidogrel with the ADP cartridge [80] but a newer cartridge called INNOVANCE PFA P2Y has shown promise in detecting clopidogrel resistance [83]. However, all aggregation assays, VHA’s, the CPA and PFA-100 are sensitive to GPIIb/IIIa inhibitors. Despite being sensitive to anti-platelet treatment the aggregometry assays selectively measures platelet function and not clot formation or fibrinolysis and Multiplate has been shown to be insensitive to factor deficiencies [84]. Both Multiplate and Plateletworks have been shown to be insensitive to fibrinogen [6,74]. The CPA in contrast is affected by fibrinogen concentration [12]. TG can be used to detect deficiencies in several coagulation factors but is less sensitive to deficiencies in fibrinogen and FXIII [69]. VHA’s are affected by fibrinogen concentration [18-20,22] as well as FXIII [23,24] which allows monitoring of treatment with fibrinogen and FXIII concentrates [23,24,55,56], although other methods are still the standard choice in the clinical setting. Haemodilution-associated coagulopathy can be detected by VHA’s [23,24,27,55,56] and aggregometry [85] but has not been detectable with TG assay [55].

A main issue to consider is the lack of flow in many of the assays (e.g. aggregometry and VHA’s) [15]. Also the ones that operate with flow vary in their shear rates such as the CPA and PFA-100 [11,12,80]. The shear can be varied in the flow chambers and can thus mimic in vivo conditions [14]. However, the perfusion chambers and CPA have low throughput, are time consuming, complicated to use and evaluate results, and require large sample volumes, although a number of new commercial flow chambers and systems are now emerging, overcoming some of these problems [15].

Many of the methods including the aggregation assays and the PFA-100 have the disadvantage that they are not suitable for samples with low platelet concentration such as patients with hematologic malignant disease and thus are not possible to use to guide prophylactic transfusions of platelets and to assess the efficacy of a transfusion in these patients. VHA’s, CPA and flow chambers have been tested in this context but further studies are warranted [47,78,86-88].

Few of the methods are recommended in guidelines, the exception is VHA’s in the European guidelines by “The multidisciplinary Task Force for Advanced Bleeding Care in Trauma” for management of bleeding and coagulopathy following major trauma where viscoelastic methods are recommended (Grade 1C) to be performed to assist in characterizing the coagulopathy and in guiding haemostatic therapy [89].

In the future, new interesting options for haemostasis testing will become available. A novel cassette-based TEG instrument is in the pipeline, as well as a better test for fibrinogen in the ROTEM (FibTEM+). Another trend is the commercialisation of flow chamber devices, either as ready-made flow cells or integrated systems with chambers, specialised pumps and software, such as the T-TAS, VenaFlux, BioFlux and Ibidi systems. The next step may be even more miniaturised, multi-channel flow cell devices, as well as devices with patterned surfaces for platelet adhesion testing. Over the past years, these devices have evolved from being able to observe shear-stress induced activation at different shear rates [90] or changing geometries [91] to devices with patterned surface coatings [92-97] and recently the combination of the two [98]. One example even includes a simple optical system for detection the onset of coagulation employing a laser and photodiode [99]. Even though this device required complex external facilities, it demonstrates one possibility for detection of coagulation in a simple point-of-care system, and more will likely follow.

The component that is missing in all current commercial devices is the endothelium, which is an important component of haemostasis in vivo, but complicated to incorporate in analyses suitable for routine use. Time will tell if addition of some components from this axis will improve the predictive power of future “global” haemostasis assays.

## Conclusions

No assay available today covers all functions of the haemostatic process. Therefore, the specific clinical question and available evidence on diagnostic performance needs to guide the choice of method. As different methods have their pros and cons, the laboratory should choose the method/s giving the most relevant information for the requesting clinician, but also one being possible to perform within the times recommended to preserve sample stability and reagent performance. Many of the assays described are still labour intensive and in their current commercial form not suitable for high-throughput sample analysis or for use and interpretation by operators with little experience. Therefore, we would only recommend them to be used in units where a sufficiently large number of samples are analysed. One interesting alternative is the possibility for some of the tests to run the samples in a central lab and display the results on a screen in the operation theatre. Another problem is to produce good control materials, as the elasticity obtained in plasma control samples are much lower than the ones that will be encountered in highly elastic samples such as blood and platelet rich plasma. Many of the tests also propose the use of single samples, something that in our opinion might be questionable considering the relatively high variability in many of the methods, a reliable use of single samples should first be verified for the actual protocol and instrument to be used. Preferably, the normal reference range to be used should also be established on site with samples collected, stored and treated in the same way as the patient samples to be analysed.
